# *Klebsiella* Phage KP34 RNA Polymerase and Its Use in RNA Synthesis

**DOI:** 10.3389/fmicb.2019.02487

**Published:** 2019-10-31

**Authors:** Xueling Lu, Hui Wu, Heng Xia, Fengtao Huang, Yan Yan, Bingbing Yu, Rui Cheng, Zuzanna Drulis-Kawa, Bin Zhu

**Affiliations:** ^1^Key Laboratory of Molecular Biophysics, the Ministry of Education, College of Life Sciences and Technology and Shenzhen College, Huazhong University of Science and Technology, Wuhan, China; ^2^Institute of Genetics and Microbiology, University of Wrocław, Wrocław, Poland

**Keywords:** T7 RNA polymerase, phiKMV, sgRNA, *in vitro* transcription, run-off RNA synthesis

## Abstract

We have characterized the single subunit RNA polymerase from *Klebsiella* phage KP34. The enzyme is unique among known bacteriophage RNA polymerases in that it recognizes two unrelated promoter sequences, which provided clues for the evolution of phage single-subunit RNA polymerases. As the first representative enzyme from the “phiKMV-like viruses” cluster, its use in run-off RNA synthesis was investigated. RNA-Seq analysis revealed that the KP34 RNA polymerase does not possess the undesired self-templated RNA terminus extension known for T7 RNA polymerase and is suitable to synthesize RNAs with structured 3′ termini such as sgRNAs. A KP34 RNA polymerase Y603F mutant is engineered to incorporate deoxy- and 2′-fluoro ribonucleotide into RNA.

## Introduction

*In vitro* run-off RNA synthesis catalyzed by phage-encoded single-subunit RNA polymerases (ssRNAP) is a fundamental technology for RNA research. The most widely used enzyme in this technique is the RNA polymerase (RNAP) encoded by bacteriophage T7 due to its high efficiency and extensive characterization (Davanloo et al., 1984; Tabor and Richardson, 1985; Milligan and Uhlenbeck, 1989). However, limitations of T7 RNAP such as RNA self-templated extension (Cazenave and Uhlenbeck, 1994; Triana-Alonso et al., 1995; Nacheva and Berzal-Herranz, 2003; Gholamalipour et al., 2018) hinder downstream RNA research and application. Therefore, it is necessary to discover novel ssRNAPs which may overcome limitations of T7 RNAP and improve *in vitro* transcription technique.

**FIGURE 1:**
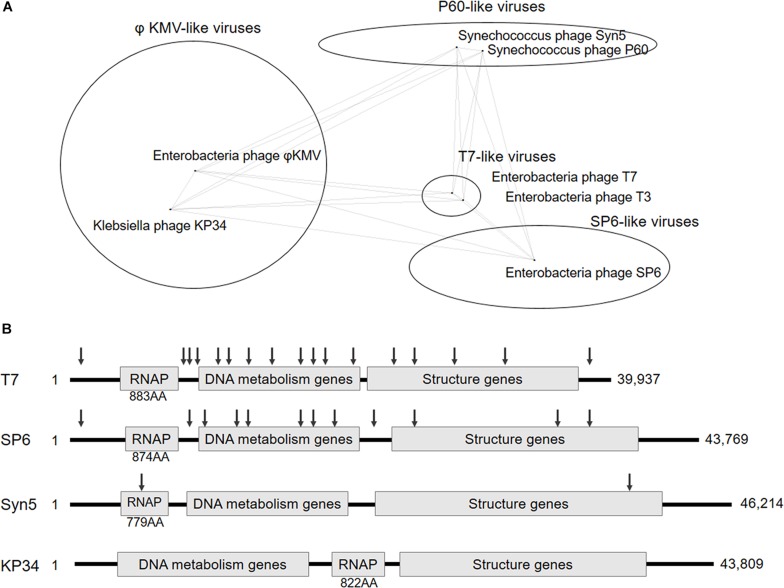
Classification and genome organization of bacteriophages containing ssRNAP. **(A)** Schematic showing of *Autographivirinae* clusters based on genome sequence comparison (an inaccurately modified version of Fig. 3 in Drulis-Kawa et al., 2011). **(B)** Schematic illustration of the genome organization of phage T7, SP6, Syn5 and KP34. The approximate position of T7, SP6, and Syn5 promoters are indicated by down arrows. Size of each RNAP and genome are also shown.

Recently blooming bacteriophage genome resources provide opportunities for new ssRNAPs discovery, and bioinformatics analysis has revealed four distinct phage clusters corresponding to T7-, SP6-, P60-, and phiKMV-like viruses in the short-tailed phage *Autographivirinae* subfamily (Drulis-Kawa et al., 2011; Figure 1A), which was known to encode ssRNAPs (Supplementary Figure S1). Among them, *Enterobacteria* phage T7 and T3 from T7-like viruses cluster, *Salmonella* phage SP6 from SP6-like viruses cluster, and *Synechococcus* phage Syn5 from P60-like viruses cluster have their ssRNAPs and transcription systems characterized (Davanloo et al., 1984; Morris et al., 1986; Krieg and Melton, 1987; Zhu et al., 2013), leaving phiKMV-like viruses the only cluster without any representative ssRNAP of its members studied.

In this work, we targeted *Klebsiella* phage KP34 to characterize the first ssRNAP from phiKMV-like viruses. Bacteriophage KP34 is a novel virus that propagates on extended-spectrum β-lactamase-producing *Klebsiella pneumoniae* strains (Drulis-Kawa et al., 2011). The genome sequence of phage KP34 consists of 43,809 bp containing 57 predicted ORFs, and the gene that encodes RNAP is between the DNA metabolism genes and the structure genes (Drulis-Kawa et al., 2011). This gene organization is typical among phiKMV-like viruses, while in all other clusters of *Autographivirinae*, the RNAP gene is located upstream of DNA metabolism genes (Figure 1B) and takes control of the transcription of the latter. The unique gene organization suggests that the ssRNAPs and transcription systems of phiKMV-like viruses are the most distant in evolution from those of the other three categories in the *Autographivirinae* subfamily, thus the characterization of KP34 RNAP may provide clues for the understanding of the evolution and mechanism of ssRNAP-based transcription systems.

With purified KP34 RNAP, we have established the KP34 *in vitro* transcription system and identified the KP34 RNAP promoter sequences. Surprisingly, four promoters divided into two unrelated classes were found within the KP34 genome, not known before for any phage ssRNAP transcription system. KP34 RNAP demonstrates its advantage over T7 RNAP in run-off RNA synthesis as it lacks the property of the latter to extend the 3′ primer/template structure of RNA (Gholamalipour et al., 2018), as revealed by RNA-Seq. The Y603F mutant of KP34 RNAP can incorporate dNMPs and 2′-fluoro-dNMPs into RNA.

## Materials and Methods

### Materials

Oligonucleotides were obtained from TsingKe (Beijing, China). DNase I, restriction endonucleases, *E. coli* inorganic pyrophosphatase, *E. coli* Poly(A) Polymerase, RNA 5′ Pyrophosphohydrolase (RppH), T4 DNA ligase, NTPs, dNTPs, and RNA purification kits were from New England BioLabs (Ipswich, MA, United States). RNase inhibitor was from Thermo Fisher Scientific (Waltham, MA, United States). PrimeSTAR Max DNA Polymerase and Premix *Taq* DNA Polymerase, SMARTScribe and ProtoScript II Reverse Transcriptase are were from TAKARA (Shiga, Japan). DNA purification kit was from Axygen (Union City, CA, United States). Ni-NTA resin was from Qiagen (Hilden, Germany). Preparative Superdex S200 for gel filtration was from GE Healthcare (Chicago, IL, United States). Radiolabeled nucleotides were from PerkinElmer (Waltham, MA, United States). 2′-F-dNTPs were from TriLink (San Diego, CA, United States).

### Protein Expression and Purification

DNA fragments encoding KP34 RNAP were amplified from the KP34 genome and inserted into pET28a vector with N-terminal His-tag. PCR amplification was performed following the user manual of PrimeSTAR Max DNA Polymerase (TAKARA). KP34 genomic DNA was prepared according to a previous report (Drulis-Kawa et al., 2011). The Plasmid pET28a-KP34 RNAP was transformed into *E. coli* BL21(DE3). The bacteria were cultured in LB medium containing 50 μg/ml kanamycin at 37°C until they reached an *A*600 of ∼1.2. The gene for KP34 RNAP was induced by the addition of 0.5 mM IPTG at 28°C and incubation continued for 3 h. Cells were harvested, resuspended and sonicated in lysis buffer (50 mM Tris–HCl, pH 8.0, 300 mM NaCl, and 20 mM imidazole). Resuspended cells were lysed via sonication on ice using 60% probe amplitude for 5 min (2 s on; 2 s off). Cell debris was pelleted by centrifugation for 50 min at 12000 rpm, 4°C. His-tagged KP34 RNAP was purified with Ni-NTA-agarose column. The lysate was run over a 2 ml (bead volume) Ni-NTA gravity column pre-equilibrated with lysis buffer. The column was washed with 10 column volumes of wash buffer (50 mM Tris–HCl, pH 8.0, 300 mM NaCl, and 50 mM imidazole). KP34 RNAP was eluted off the column by 4 column volumes of elution buffer (50 mM Tris–HCl, pH 8.0, 300 mM NaCl, and 200 mM imidazole). The RNAP was further purified by gel filtration chromatography on a 200 ml preparative Superdex S200 column. The column was pre-equilibrated with a 400–500 ml balance buffer (50 mM Tris–HCl, pH 8.0, 100 mM NaCl, 0.1 mM EDTA and 1 mM DTT). Then a 1–2 ml sample which was concentrated by Millipore Amicon Ultra-15 (30,000 MWCO) was added to the column. Fractions were eluted off the column by 200–300 ml balance buffer. Fractions containing the KP34 RNAP were concentrated again and dialyzed twice against a storage buffer (50 mM Tris–HCl, pH 7.5, 100 mM NaCl, 0.1 mM EDTA, 1 mM DTT, 50% glycerol, and 0.1% Triton^TM^ X-100). KP34 RNAP mutations were introduced through PCR mutagenesis using PrimeSTAR Max DNA Polymerase, and mutant RNAPs were purified following the same procedure as that for the wild-type enzyme. The T7 RNAP gene was cloned into plasmid pQE-82L through homologous DNA assembly using ClonExpress^®^ II One Step Cloning Kit from Vazyme (Nanjing, China). The plasmid pQE-82L-T7 RNAP was transformed into BL21(DE3). N-terminal His-tagged T7 RNAP was purified following the same procedure as mentioned above for KP34 RNAP. His-tagged Syn5 RNAP was produced from a pET24 vector harboring the N-terminal His-tagged Syn5 RNAP gene between the *Nde*I and *Not*I sites and purified according to previous report (Zhu et al., 2015). Oligonucleotide primers used for cloning and mutagenesis are listed in Supplementary Table S1. Protein concentration was determined by the Bradford method, and all RNA polymerases tested in this work were analyzed on SDS-PAGE gel (Supplementary Figure S2).

### DNA Templates

**FIGURE 2:**
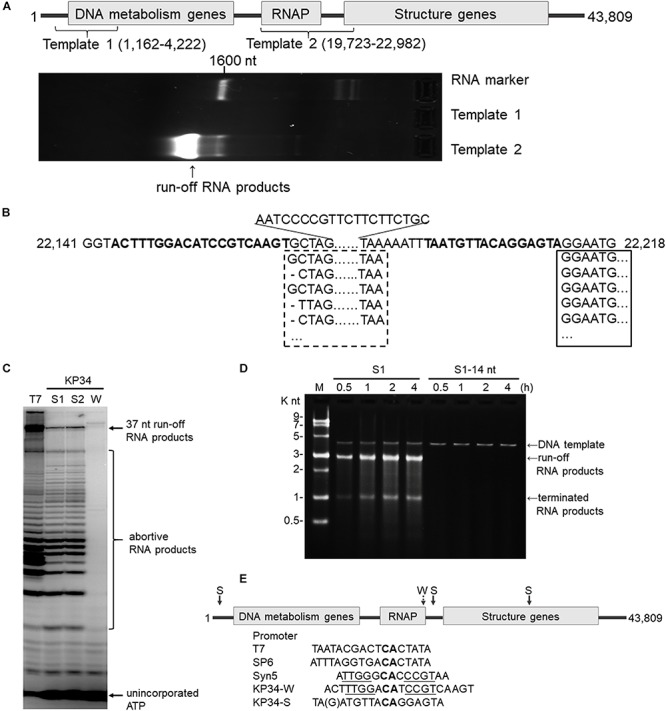
Identification of the KP34 RNAP promoter. **(A)** The DNA fragment (Template 1) containing previously predicated promoters failed to serve as transcription template for KP34 RNAP to produce RNA *in vitro*, while the DNA fragment covering the RNAP gene and downstream gap region (Template 2) was active as transcription template. RNA products are shown as bright bands on the 2% TAE agarose gel. **(B)** 5′-RACE analysis of the position of transcription initiation. 5′ sequence of KP34 transcripts were matched to KP34 genome. Major sequences were in solid box and minor sequences in dotted box. Their upstream region containing putative promoters is shown in bold. **(C)** Comparison of run-off RNA synthesis by T7 and KP34 RNAP under the control of various promoters. A DNA template containing a T7 promoter (5′-TAATACGACTCACTATA-3′) was incubated with 100 nM T7 RNAP, and three DNA templates containing either a KP34 strong promoter S1 (5′-TAATGTTACAGGAGTA-3′), a KP34 strong promoter S2 (5′-TGATGTTACAGGAGTA-3′), or a KP34 weak promoter W (5′-ACTTTGGACATCCG TCAAGT-3′) were incubated with 100 nM KP34 RNAP to direct to the transcription of their downstream sequence that encodes the same 37 nt RNA. [α-32P]ATP was added into reactions for imaging and visualization. Reaction products were separated by a 25% TBE-Urea denaturing gel. **(D)** Identification of the full KP34 strong promoter. A KP34 strong promoter S1 (5′-TAATGTTACAGGAGTA-3′) or the 3′ 14 nt common sequence of the two KP34 strong promoters (5′-ATGTTA CAGGAGTA-3′) was inserted into plasmid pUC19 to direct the transcription of their downstream sequences, respectively. A run-off transcript of ∼2700 nt and a terminated transcript of ∼1000 nt (terminated by a predictable T7 class I hairpin terminator structure) were expected from the linearized form of these plasmids if the inserted promoter is sufficient to direct transcription by KP34 RNAP. M: ssRNA Ladder. **(E)** KP34 promoters in the genome (location of strong promoter (S) pointed by solid arrow and weak promoter (W) by dotted arrow) and comparison of typical ssRNAP promoters. Conserved sequence among ssRNAP promoters are in bold and those homologous between Syn5 promoter and KP34 weak promoter are underlined.

**FIGURE 3:**
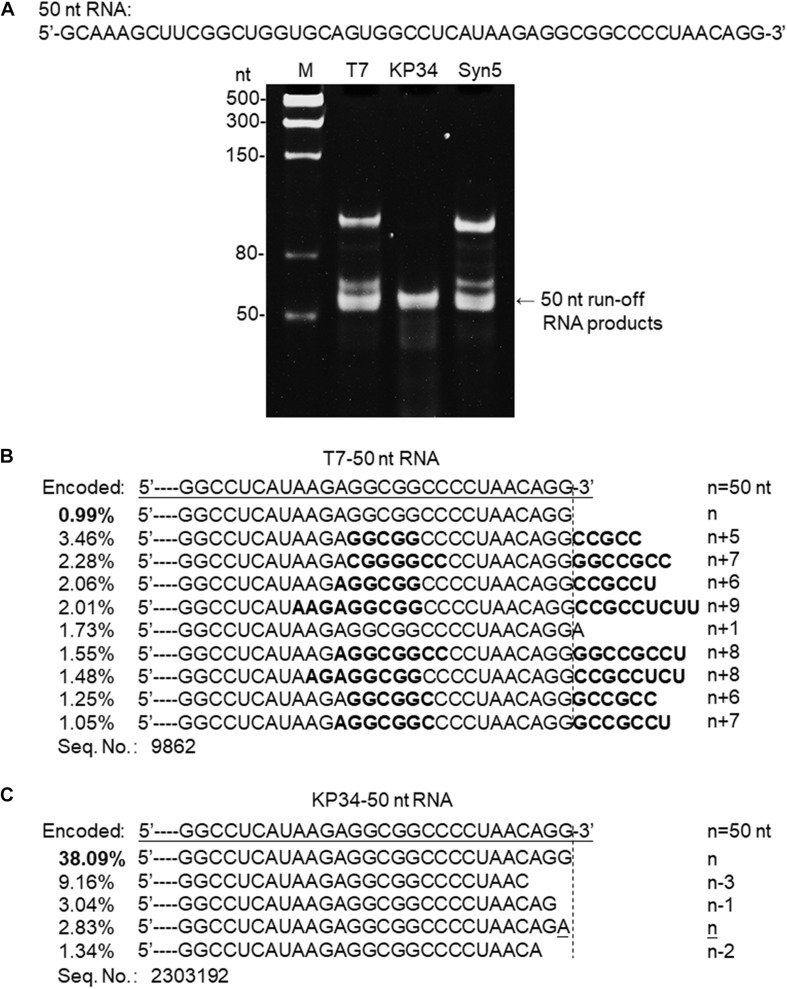
Synthesis of a 50 nt RNA containing 3′ hairpin structure by various RNAPs. **(A)** The 50 nt RNA sequence is shown at the top of the gel. The three DNA templates containing the same coding sequences for the 50 nt run-off RNA transcripts under the control of either a T7 promoter, a KP34 strong promoter, or a Syn5 promoter were incubated with 0.2 μM T7 RNAP, 1 μM KP34 RNAP, or 1 μM Syn5 RNAP, respectively. Incubation with KP34 and T7 RNAP was at 37°C for 1 h and incubation with Syn5 RNAP was at 24°C for 1 h. Reaction products were separated by a 12% TBE native gel and then stained with ethidium bromide. M: ssRNA Ladder. **(B)** RNA-Seq analysis of the 3′ termini of T7 RNAP transcripts. 3′ termini of sequences with reads more than 1% of total reads were aligned and shown. Percentage of major sequences in total sequencing results are noted and percentage of the correct product is in bold. A dotted line cut indicates the precise terminus encoded by DNA template, and the number of extended nt is shown as n + x. Bold sequences indicate complementary sequences in each RNA specie resulted from extension of a possible 3′ self-primed structure. **(C)** Similar as B, RNA-Seq analysis of the 3′ termini of KP34 RNAP transcripts. Number of missing nt at the 3′ terminus of major sequences is shown as n–x.

**FIGURE 4:**
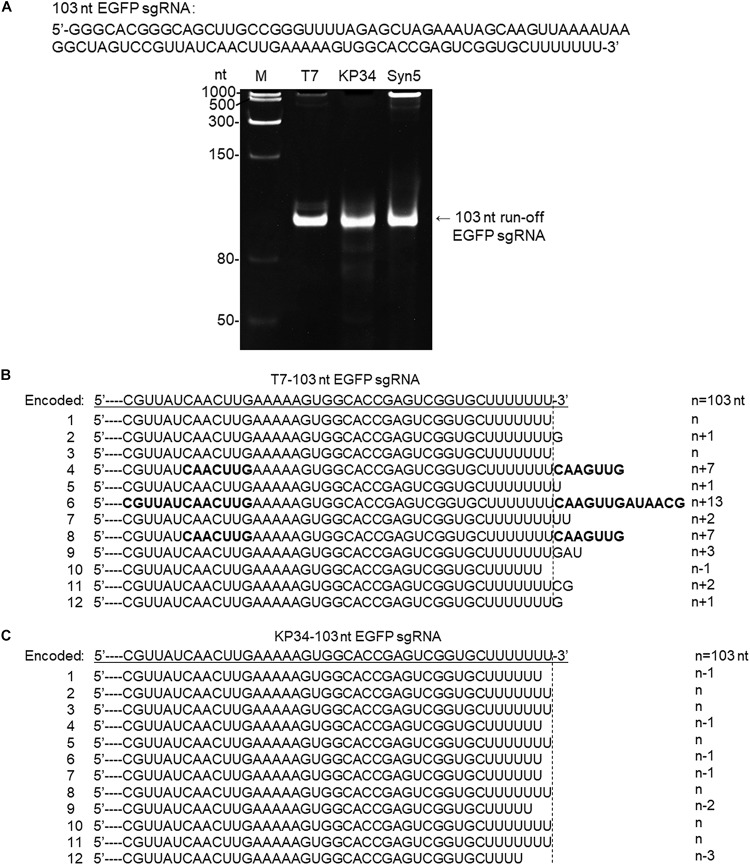
Synthesis of an sgRNA by T7 and KP34 RNAP. **(A)** The sgRNA sequence is shown at the top of the gel. The three DNA templates containing the same coding sequences for the sgRNA under the control of either a T7 promoter, a KP34 strong promoter, or a Syn5 promoter were incubated with 0.2 μM T7 RNAP, 1 μM KP34 RNAP, or 1 μM Syn5 RNAP, respectively. Incubation with KP34 and T7 RNAP was at 37°C for 1 h and incubation with Syn5 RNAP was at 24°C for 1 h. Reaction products were separated by a 12% TBE native gel and then stained with ethidium bromide. M: ssRNA Ladder. **(B)** 3′-RACE analysis of the 3′ termini of T7 RNAP transcripts. 3′ termini of obtained sequences were aligned and shown. A dotted line cut indicates the precise terminus encoded by DNA template and number of extended or missing nt is shown as n + x or n–x. Bold sequences indicate complementary sequences in each RNA resulted from extension of possible 3′ self-primed structures. **(C)** Similar as B, 3′-RACE analysis of the 3′ termini of KP34 RNAP transcripts.

**FIGURE 5:**
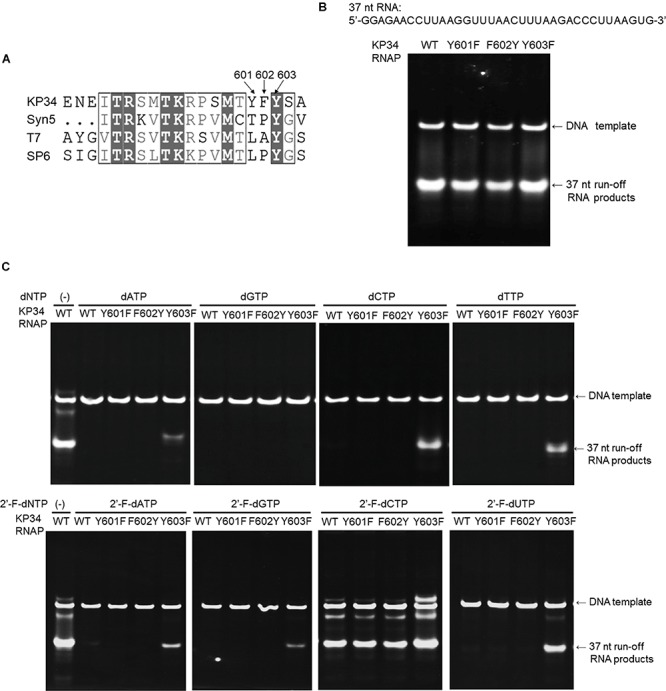
Incorporation of dNMPs and 2′-F-dNMP by wild-type (WT) KP34 RNAP and its Y601F, F602Y, and Y603F mutants. All RNA polymerases in the assays were at the same final concentration 1 μM. Transcription reaction mixtures were separated by 12% TBE native gel and then stained with ethidium bromide. **(A)** KP34-Y601F, F602Y and KP34-Y603F RNAPs were constructed based on Multiple Sequence Alignment by ClustalW to the region containing either Y639 in T7 RNAP, Y631 in SP6 RNAP or Y564 in Syn5 RNAP, which were known to control the ribose 2′ specificity of each enzyme. **(B)** Comparison of WT, Y601F, F602Y, and Y603F KP34 RNAPs in synthesis of natural RNA. The 37 nt RNA sequence to be synthesized is at the top of the gel. The enzyme used for each reaction is indicated at the top of each lane. **(C)** Incorporation of dNMPs and 2′-F-dNMPs into the 37 nt RNA transcripts by WT, Y601F, F602Y, and Y603F KP34 RNAPs. In each reaction, one of the four normal NTP substrates was replaced by the corresponding dNTP or 2′-F-dNTP analog as indicated at the top of the gels.

Transcription templates for Figures 2C, 3, 5 were prepared by annealing two complementary synthetic DNA oligonucleotides, respectively. Transcription templates for Figure 2A were PCR amplified from the KP34 genomic DNA. To prepare transcription templates for Figure 2D, plasmid pUC19 with a 16 nt or 14 nt KP34 promoter sequence plus three consecutive guanosine residue (in bold) (5′-TAATGTTACAGGAGTA**GGG**-3′ or 5′-ATGTTACAGGAGTA**GGG**-3′) insertion between the *Bam*HI and *Xba*I sites was linearized by *Eco*RI treatment. For Figure 4, the coding sequence for an EGFP sgRNA (5′-GGGCACGGGCA GCTTGCCGGGTTTTAGAGCTAGAAATAGCAAGTTAAAAT AAGGCTAGTCCGTTATCAACTTGAAAAAGTGGCACCGAG TCGGTGCTTTTTTTTGAAGAGC-3′) under the control of a T7 promoter (5′-TAATACGACTCACTATA-3′), a KP34 promoter (5′-TAATGTTACAGGAGTA-3′), or a Syn5 promoter (5′-ATTGGGCACCCGTAA-3′) was inserted into the plasmid pUC19 between the *Bam*HI and *Xba*I sites, and transcription templates were then PCR amplified from the plasmids carrying each of the three promoters, respectively. Sequences of all PCR primers and synthetic DNA oligonucleotides mentioned above were listed in Supplementary Table S2. PCR reactions for DNA templates were carried out using PrimeSTAR Max DNA Polymerase according to the manual from the manufacturer (TAKARA). PCR products and linearized plasmids were purified using AxyPrep^TM^ PCR Cleanup Kit (Axygen), and DNA concentrations were determined by NanoPhotometer^®^ (Implen).

### Transcription Assays

For the assays shown in Figures 2A,D, 3A, 4A, 5B,C, the transcription reaction mixtures (10 μl) contained 40 mM Tris–HCl (pH 8.0), 2 mM spermidine, 20 mM DTT, 20 mM MgCl_2,_ 4 mM of each of the 4 NTPs (with 0 to 4 of the NTPs replaced by their dNTP or 2′-F-dNTP analogs), 40 U/μl RNaseOUT^TM^ Recombinant Ribonuclease Inhibitor, 0.04 U/μl *E. coli* inorganic pyrophosphatase, DNA template (2 μM annealed oligonucleotides, 20 nM linearized plasmids, or 0.5 μM PCR products), and RNA polymerase (1 μM KP34 RNAP or its mutants, or 0.2 μM T7 RNAP or 1 μM Syn5 RNAP). Reactions with KP34 and T7 RNAP were carried out at 37°C for 1 h and reactions with Syn5 RNAP were carried out at 24°C for 1 h. For the assays shown in Figures 2A, 3A, 4A, one unit of DNase I was added to each reaction mixture and incubated for an additional 20 min at 37°C to remove the DNA templates. A total of 1 μl of the reaction mixture was then mixed with 9 μl deionized H_2_O and 5 μl 3X RNA loading dye (95% formamide, 40 mM EDTA, 0.025% bromophenol blue and 0.025% Xylene cyanol FF). Then, 7 μl of each sample was loaded onto 12% TBE native gels or 2% TAE agarose gels. DNA templates and RNA products were separated by electrophoresis and visualized by staining with ethidium bromide. The transcripts were purified with Monarch^®^ RNA Cleanup Kit (New England BioLabs) and their concentration was determined using NanoPhotometer^®^(Implen). The reaction mixtures for Figure 2C contained 40 mM Tris–HCl (pH 8.0), 6 mM MgCl_2_, 2 mM spermidine, 10 mM DTT, 200 μM CTP, GTP and UTP, 10 μM [α-32P]ATP, 1.5 U/ml RNaseOUT^TM^ recombinant ribonuclease inhibitor, 0.5 μM DNA templates, and 100 nM KP34 or T7 RNAP that were incubated at 37°C for 30 min before adding one unit of DNase I to each reaction mixture, and then incubated for an additional 20 min at 37°C. Reactions were then terminated by the addition of 4 μl of 3X RNA loading dye containing 95% formamide, 40 mM EDTA, 0.025% bromophenol blue and 0.025% Xylene cyanol FF. Samples were then heated at 90°C for 1 min, and 4 μl of each sample was loaded onto a 25% TBE-Urea denaturing gel. After electrophoresis, gels were dried and the radioactivity was analyzed using a Fuji BAS 1000 Bioimaging Analyzer.

### RNA-Seq

*In vitro* transcription products were treated with DNase I for 20 min at 37°C to remove the DNA templates and then were purified with Monarch^®^ RNA Cleanup Kit (New England BioLabs). Transcripts were incubated with RNA 5′ Pyrophosphohydrolase (RppH) at 37°C for 30 min to remove pyrophosphate from the 5′ end of triphosphorylated RNAs and to leave 5′ monophosphate. 5′ monophosphorylated RNAs were purified with Monarch^®^ RNA Cleanup Kit (New England BioLabs), and the cDNA library was prepared by the NEBNext Small RNA Library Prep Set for Illumina (New England BioLabs). The cDNA library was sequenced on an Illumina HiSeq 2500 sequencing platform. The raw data in FASTQ format was obtained from the SeqHealth company (Wuhan, China).

### RNA 5′- and 3′-RACE

Transcription reactions (10 μl) were incubated at 37°C for 20 min with DNase I to remove the DNA templates. Transcripts were purified with a Monarch^®^ RNA Cleanup Kit (New England BioLabs) and then incubated with *E. coli* Poly(A) Polymerase at 37°C for 30 min to add adenosines to the 3′ end of RNA. The products were purified again. RNAs were then reverse transcribed to cDNA.

5′-RACE: 1–3.5 μl RNA (1 ng–1 μg of poly A^+^ RNA) was mixed with 1 μl 3′ SMART CDS Primer II A (12 μM) to 4.5 μl total volume. The sample RNA/primer was denatured for 5 min at 65°C and was then spun briefly and put promptly on ice. The following components were added (5.5 μl): 2 μl 5X First-Strand Buffer, 0.25 μl DTT (100 mM), 1 μl dNTP Mix (10 mM), 1 μl SMARTer II A Oligonucleotide (12 μM), 0.25 μl RNase Inhibitor (40 U/μl), and 1 μl SMARTScribe Reverse Transcriptase (100 U/μl). Reaction mixtures were incubated at 42°C for 1 h. cDNA amplification was done by 12.5 μl Premix *Taq* DNA Polymerase (TAKARA) using 0.2 μM 5′ universal prime (UPM) and 0.2 μM 3′ gene specific primer (GSP1) in a 25 μl reaction volume. 3′-RACE: 100 ng poly A^+^ RNA, 5 μM 2 μl VN-oligo(dT)_18_(50 μM) was mixed with 1 mM dNTP to 10 μl total volume. Sample RNA/primer was denatured for 5 min at 65°C, then spun briefly and put promptly on ice. The following components were added (10 μl):4 μl 5X ProtoScript II Buffer, 2 μl 0.1 M DTT, 1 μl ProtoScript II Reverse Transcriptase (200 U/μl), 0.2 μl RNase Inhibitor (40 U/μl), and 2.8 μl deionized H_2_O. The 20 μl cDNA synthesis reaction was incubated at 42°C for 1 h. cDNA was then amplified by 12.5 μl Premix *Taq* DNA Polymerase (TAKARA) using 0.2 μM 5′ gene specific primer (GSP2) and 0.2 μM 3′ VN primer in a 25 μl reaction volume.

PCR products were purified by AxyPrep^TM^ PCR Cleanup Kit, and then ligated to T-Vector pMD19 with T4 DNA ligase (2X ligation Mix); the reaction was incubated at 16°C for 30 min. Plasmids containing inserted sequences were amplified and sent for sequencing.

## Results and Discussion

### *Klebsiella* Phage KP34 and Its RNAP

*Klebsiella* phage KP34 is distantly related to the T7-, SP6- and P60-like viruses of the short-tailed phage *Autographivirinae* subfamily (Figure 1A) and belongs to the cluster of phiKMV-like viruses, which is the only phage cluster in the subfamily without any RNAP of its member characterized. Predicted KP34 RNA polymerase consists of 822 amino acids (AA), which is larger in size than Syn5 RNAP (779 AA) and smaller than T7 (883 AA) and SP6 RNAP (874 AA), and compared to characterized phage ssRNAPs it is the only one with its coding gene located downstream of the DNA metabolism genes (Figure 1B). KP34 RNAP with over 90% homogeneity was purified as N-terminal His-tagged protein (Supplementary Figure S2).

### Identification of the KP34 RNAP Promoter

Bioinformatics has predicted KP34 transcription promoter to be a consentaneous sequence 5′-AGCCTATA GCGTCCTACGGGGCGCTATGTGAA-3′ in the early region of the phage genome (Eriksson et al., 2015), because it is the only sequence that is longer than 20 nt and appears twice within the genome. However, a DNA fragment (template 1) made by PCR amplification of this region failed to serve as the template for transcription by KP34 RNAP to produce any RNA product *in vitro* (Figure 2A).

Deduced from the promoter distribution of known phage ssRNAP promoters (Figure 1B), we predicted that there should be at least one KP34 promoter in the region covering the RNAP gene [as is the case for Syn5 (Zhu et al., 2013)] and the intergenic region between the RNAP and the next gene [as the cases for T7 and SP6 (Davanloo et al., 1984; Krieg and Melton, 1987; Chen and Schneider, 2005)]. Indeed, DNA template 2 made accordingly directed the production of RNA transcripts by KP34 RNAP (Figure 2A). 5′-RACE revealed that most of these transcripts initiated at nucleotide (nt) 22213 in the genome (Figure 2B), strongly suggesting that its upstream region, right behind the RNAP gene (nt 19723–22191), contains the major KP34 promoter. A 16 nt sequence 5′-TAATGTTACAGGAGTA-3′ was identified in this region by sequence homology analysis; it appears twice in KP34 genome (nt 22197–22212, 33721–33736) and differs with another sequence 5′-TGATGTTACAGGAGTA-3′ (475-490) by only the 5′ second nt. *In vitro* transcription assay confirmed that these two 16 nt sequences promote KP34 RNAP transcription at similar efficiency (Figure 2C, S1 vs. S2), while the common 14 nt sequence of these two is much less efficient as KP34 promoter (Figure 2D). Thus 5′-T(A/G)ATGTTACAGGAGTA-3′ was designated as KP34 strong promoter. To our surprise, 5′-RACE also identified a small portion of transcripts not initiated by the strong promoter. These RNA 5′ sequences match the 3′ region (∼nt 22164) of a putative proximal weak promoter within the RNAP gene (Figure 2B). The 5′ termini of RNA from this promoter show high heterogeneity (Figure 2B), indicating that it is an inefficient and inaccurate promoter. *In vitro* transcription assay confirmed that the upstream region containing the sequence 5′-ACTTTGGACATCCGTCAAGT-3′ (nt 22144–22163) also initiates KP34 RNAP transcription, while with much less efficiency and precision, as judged by the amount and heterogeneity of run-off products (Figure 2C, W vs. S1 or S2). Thus, this sequence was designated as KP34 weak promoter. Coincidentally, the *Synechococcus* phage Syn5 promoter is also located within its RNAP gene and shares sequence similarity with KP34 weak promoter (Figure 2E). Like other phage ssRNAPs, KP34 RNAP also generates large amounts of abortive transcripts ranging from a few to more than 20 nt during transcription, especially under the control of its strong promoter (Figure 2C), likely due to the transition from transcription initiation to elongation. However, it was noticed that the abortive transcript distributions are different between KP34 and T7 RNAP (Figure 2C), of which the underlying mechanism is to be explored.

The whole picture of representative phage ssRNAP systems was filled by the characterization of KP34 RNAP and its promoters (Figures 1B, 2E). Some phages (such as Syn5) retained their major promoter associated with the RNAP gene to fulfill their transcription, while others (such as T7 and SP6) have evolved promoters at multiple genome locations to control transcription in a finer regulated manner. As an intermediate status, KP34 has evolved its specific strong promoter and may rely only on them for transcription control, but its promoter associated with the RNAP gene, which is likely the primitive type, still remains as an evolutionary relic.

To develop the use of KP34 RNAP *in vitro* transcription, we optimized its reaction condition in preparative scale RNA synthesis (Supplementary Figures S3–S6). The highest yield of run-off RNA in these assays was observed at a temperature of 37°C–42°C (Supplementary Figure S3), pH 7.5–8.5 (Supplementary Figure S4), and a MgCl_2_ concentration of 10–20 mM (Supplementary Figure S5). MnCl_2_ with an optimized concentration of 10 mM also supports KP34 RNAP transcription but is less efficient compared to MgCl_2_ (Supplementary Figure S5). Addition of salts NaCl, KCl or NHCl_4_ in the reaction all showed inhibitory effect (Supplementary Figure S5). Thus, for convenience we set 37°C, pH 8.0 and 20 mM MgCl_2_ as standard KP34 RNAP reaction conditions.

### KP34 RNAP Does Not Catalyze RNA Self-Templated 3′ End Extension

SsRNAPs are fundamental enzymatic tools for the production of RNA for research and application, due to their simplicity and high efficiency, while some of their undesired properties may affect the quality of RNA products and hinder downstream RNA application. For example, the most popular ssRNAP for *in vitro* transcription-T7 RNAP catalyzes undesired RNA 3′ end extension through multiple mechanisms [N + 1 (Milligan et al., 1987; Krupp, 1988), template switch (Rong et al., 1998) and self-primed extension (Triana-Alonso et al., 1995; Nacheva and Berzal-Herranz, 2003)]. Recent analysis based on RNA-Seq clearly demonstrated the severe product 3′ heterogeneity from T7 RNAP reactions caused by self-templated extension on the intermolecular or intramolecular primer/template-like RNA structures (Gholamalipour et al., 2018). We have encountered the same problem when synthesizing a 50 nt RNA 5′-GCAAAGCUUCGGCUGGUGCAGUGGCC UCAUAAGAGGCGGCCCCUAACAGG-3′ derived from an RNA virus genome. Both T7 and Syn5 RNAP produce highly heterogenous products (Figure 3A). However, the products of KP34 RNAP appear to form a uniform band in the gel (Figure 3A), missing the bands corresponding to products of larger size as those observed with T7 and Syn5 RNAP. To identify the transcription products by T7 and KP34 RNAP, RNA-Seq was performed. In T7 products, RNA with exactly the desired sequence accounts for surprisingly less than 1% (Figure 3B), while most products (all sequences with reads more than 1% of total reads were listed in Figure 3B) have their 3′ ends extended with several extra nts that are complementary to part of the RNA inner sequence, indicating that they are formed by the extension of the self-templated terminal structure. However, in major KP34 RNAP products (all sequences with reads more than 1% of total reads were listed in Figure 3C), ∼38% are correct sequences without any extension at the 3′ ends. On the contrary, most incorrect products are missing 1–3 nt at their 3′ ends. It is not clear yet whether these truncations are caused by the RNAP activity or environmental RNase degradation.

To confirm that KP34 RNAP is advantageous over T7 RNAP for the synthesis of RNA with 3′ secondary structure, we compared the synthesis of another such RNA, the recently widely used single-guide RNA (sgRNA for CRISPR/Cas9 based genome editing) by the two RNAPs. In gel analysis of an sgRNA synthesis, as expected, a product larger than that of the desired size was not observed for KP34 RNAP while T7 and Syn5 RNAP products contain species significantly larger in size, indicating RNA 3′ undesired extension by these RNAPs and further formation of complex structures by the extended RNA (Figure 4A). Further verified by 3′-RACE, various 3′ overextended (up to 13 nts) RNA species were found in T7 RNAP products (Figure 4B), while only 3′ truncated RNA found in KP34 products (Figure 4C). Considering there are several protecting nts at the 3′ end of sgRNA, the truncated RNA products should interfere with genome editing application less than the overextended products since the overextension may disturb the functional structure at the 3′ end of sgRNA, thus KP34 is especially suitable to synthesize sgRNA.

### Incorporation of dNMPs and 2′-Fluoro-dNMPs (2′-F-dNMP) by KP34 RNAP and Its Mutants

RNA containing ribose modifications such as 2′-F is more resistant to RNase A, the most common RNase contaminant, which recognizes the 2′-OH of pyrimidines for cleavage, resulting in a longer survival time of 2′-F-RNA *in vitro* and *in vivo* (Pieken et al., 1991; Ono et al., 1997; Sabahi et al., 2001; Pallan et al., 2011). RNAPs usually prefer NTPs strongly against dNTPs and 2′-F-dNTPs; however, replacement of a tyrosine with a phenylalanine in the “O-helix” region such as those in the T7 Y639F (Sousa and Padilla, 1995; Padilla and Sousa, 1999) and Syn5 Y564F (Zhu et al., 2015) RNAP mutants can weaken this discrimination and allow synthesis of partially modified 2′-F-RNA.

To broaden the application of KP34 RNAP, we investigated the effect of such mutations in the enzyme. Sequence alignment of the KP34 RNAP, Syn5 RNAP, T7 RNAP, and SP6 RNAP was performed with Clustal Omega (Sievers et al., 2011). The alignment was processed for publication using the ESPRIPT server v 3.0 (Robert and Gouet, 2014; Supplementary Figure S1). Interestingly, KP34 RNAP possesses three consecutive aromatic amino acids Y601/F602/Y603 at the homologous position (Figure 5A), unique among known ssRNAPs. Correspondingly, we constructed Y601F, F602Y, and Y603F mutants of KP34 RNAP and obtained purified enzymes (Supplementary Figure S2). Tested on the synthesis of a 37 nt RNA, we found that it is the Y603 that plays a significant role in controlling the ribose 2′ specificity of KP34 RNAP, as the Y603F mutant can utilize almost all sets of dNTPs and 2′-F-dNTPs as substrates and incorporate dNMPs and 2′-F-dNMPs into RNA while retaining a similar efficiency for NMP incorporation as the wild-type enzyme (Figures 5B,C). Although no production of RNA was detected with dGTP (likely because G is the initiating nt), the Y603F can even initiate RNA synthesis with 2′-F-dGTP (Figure 5C). Except for 2′-F-dCMP, no dNMP or 2′-F-dNMP incorporation was detected for Y601F and F602Y mutants. Both the wild-type and mutant enzymes incorporate 2′-F-dCMP efficiently (Figure 5C). The KP34 RNAP Y603F also incorporates 2′-F-dNMPs into sgRNA, making it suitable for the synthesis of 2′-F-sgRNA (Supplementary Figure S7). It was noticed that upon incorporation of some dNMPs or 2′-F-dNMPs, the mobility of RNA in native gels shifted significantly (Figure 5C and Supplementary Figure S7), which is likely due to the ribose 2′ modification that alters the molecular weight and the structure of the RNA. However, the possibility that the ribose 2′ modifications may also affect the behavior of the KP34 RNA polymerase at the termini of its transcripts, for example, inducing 3′ extension, remains to be clarified.

## Data Availability Statement

RNA sequencing data have been deposited in the Sequence Read Archive (SRA) (http://www.ncbi.nlm.nih.gov/sra) with the BioProject accession code PRJNA555705.

## Author Contributions

XL and BZ designed and performed the experiments and wrote the manuscript. HW and HX analyzed the data. FH guided protein expression and purification. YY, BY, RC, and ZD-K provided reagents and contributed to manuscript revision.

## Conflict of Interest

The authors declare that the research was conducted in the absence of any commercial or financial relationships that could be construed as a potential conflict of interest.
