# Supplementary material for: Klebsiella Phage KP34 RNA Polymerase and Its Use in RNA Synthesis
Source: Front Microbiol. 2019 Oct 31;10:2487. doi: 10.3389/fmicb.2019.02487 (PMC6834552; doi:10.3389/fmicb.2019.02487)
Supplement: Supplementary file 1 [file Data_Sheet_1.PDF]

Supplementary Materials

Supplementary Table 1. Oligonucleotide primers used for cloning and mutagenesis in this work.

Genes	Primers*	Sequences (5'-3')
KP34 RNAP	F	ACTG**GCTAGC**ATGATTAGCGCCCTAAGTACGGTAGTAGTACC
	R	ACTG**GCGGCCGC**TTAGCAGAAGAAGAACGGGGATTCTAGCACTTG
T7 RNAP	F	TCACCATCACCATCACCATATGAACACGATTAACATCGCTAAGA
	R	AGTCCAAGCTCAGCTAATTTTACGCGAACGCGAAGTCCGACTCT
pQE-82L	F	AATTAGCTGAGCTTGGACTCCTGTTGATAG
	R	ATGGTGATGGTGATGGTGAGATCCTCTCAT
KP34-Y601F RNAP	F	GTATGACCTTCTTCTACAGCGCCACGG
	R	CTGTAGAAGAAGGTCATACTGGGGCGC
KP34-F602Y RNAP	F	TGACCTACTACTACAGCGCCACGGTGC
	R	GCGCTGTAGTAGTAGGTCATACTGGGG
KP34-Y603F RNAP	F	CCTACTTCTTCAGCGCCACGGTGCGTA
	R	GTGGCGCTGAAGAAGTAGGTCATACTG

Introduced restriction enzyme sites are indicated in bold.
*F refers to forward primer and R refers to reverse primer.

Supplementary Table 2. DNA templates used for *in vitro* transcription assays in this work.

DNA templates	Oligos*	Sequences (5'-3')	Usage
Template 1	F	GGTTCCACGGTAGTGC<br>AGTGGG	Figure 2A<br>Primers to amplify<br>PCR fragments
	R	CACTACATCATCGCCCT<br>CATAG	
Template 2	F	ACTGGCTAGCATGATT<br>AGCGCCCTAAGTACGG<br>TAGTAGTACC	Figure 2A<br>Primers to amplify<br>PCR fragments
	R	GACGCCTGCGTCCTGG<br>TCCTTC	
T7-37	F	**TAATACGACTCACTAT**<br>**A**GGAGAACCTTAAGGT<br>TTAACTTTAAGACCCTT<br>AAGTG	Figure 2C<br>Annealed<br>oligonucleotides<br>as template
	R	CACTTAAGGGTCTTAA<br>AGTTAAACCTTAAGGT<br>TCTCCTATAGTGAGTCG<br>TATT	
KP34-S1-37	F	T**TAATGTTACAGGAGT**<br>**A**GGAGAACCTTAAGGT<br>TTAACTTTAAGACCCTT<br>AAGTG	Figure 2C<br>Annealed<br>oligonucleotides<br>as template
	R	CACTTAAGGGTCTTAA<br>AGTTAAACCTTAAGGT<br>TCTCCTACTCCTGTAAC<br>ATTAA	
KP34-S2-37	F	T**TGATGTTACAGGAGT**<br>**A**GGAGAACCTTAAGGT<br>TTAACTTTAAGACCCTT<br>AAGTG	Figure 2C<br>Annealed<br>oligonucleotides<br>as template
	R	CACTTAAGGGTCTTAA<br>AGTTAAACCTTAAGGT<br>TCTCCTACTCCTGTAAC<br>ATCAA	
KP34-W-37	F	T**ACTTTGGACATCCGT**<br>**CAA**GTGGAGAACCTTA<br>AGGTTTAACTTTAAGA<br>CCCTTAAGTG	Figure 2C<br>Annealed<br>oligonucleotides

	R	CACTTAAGGGTCTTAAAGTTAAACCTTAAGGTTCTCCACTTGACGGATGTCCAAAGTA	as template
T7-50	F	**TAATACGACTCACTATA**GCAAAGCTTCGGCTGGTGCAGTGGCCTCATAAGAGGCGGCCCCTAACAGG	Figure 3A<br>Annealed oligonucleotides as template
	R	CCTGTTAGGGGCCGCCTCTTATGAGGCCACTGCACCAGCCGAAGCTTTGCTATAGTGAGTCGTATTA	
KP34-50	F	T**TAATGTTACAGGAGTA**GCAAAGCTTCGGCTGGTGCAGTGGCCTCATAAGAGGCGGCCCCTAACAGG	Figure 3A<br>Annealed oligonucleotides as template
	R	CCTGTTAGGGGCCGCCTCTTATGAGGCCACTGCACCAGCCGAAGCTTTGCTACTCCTGTAACATTAA	
Syn5-50	F	T**ATTGGGCACCCGTAA**GCAAAGCTTCGGCTGGTGCAGTGGCCTCATAAGAGGCGGCCCCTAACAGG	Figure 3A<br>Annealed oligonucleotides as template
	R	CCTGTTAGGGGCCGCCTCTTATGAGGCCACTGCACCAGCCGAAGCTTTGCTTACGGGTGCCCAATA	
EGFP sgRNA	F	CATATGCGGTGTGAAATACCGCACAGATGC	Figure 4A<br>Primers to amplify PCR fragments
	R	AAAAAAAGCACCGACTCGGTGCCACTTTTTCAAG	

The promoter sequences are indicted in bold and the nucleotides corresponding to the run-off RNA sequences are underlined.

*F indicates forward primers (for PCR fragments) or sense strands (for annealed oligos) while R indicates reverse primers (for PCR fragments) or antisense strands (for annealed oligos).

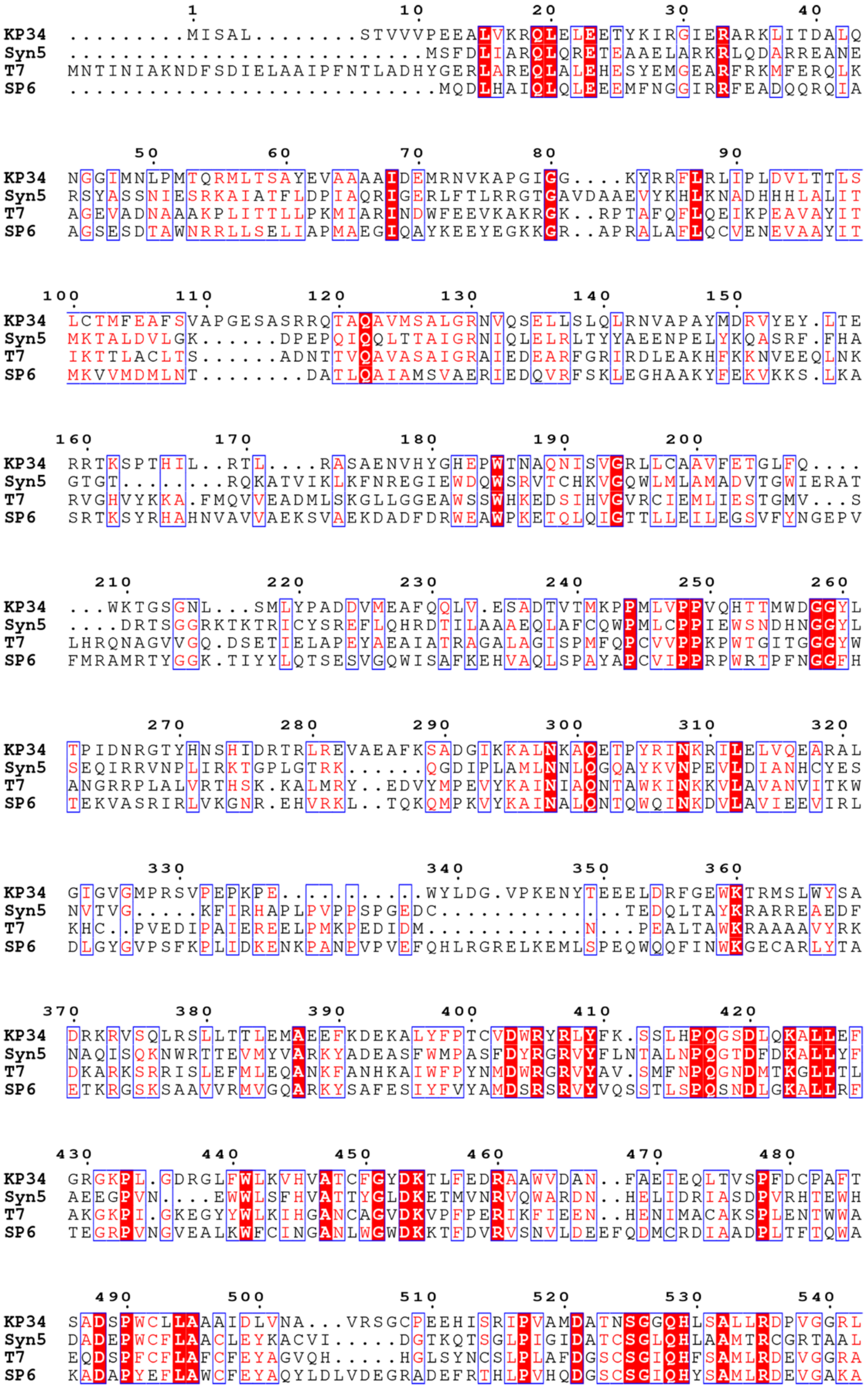
1 10 20 30 40
KP34 ....MISAL........STVVVPEEALVKRQLELEETYKIRGIERARKLITDALQ
Syn5 .........................MSFDLIARQLQRETEAAELARKRLQDARREANE
T7 MNTINIAKNDFSDIELAAIPFNTLADHYGERLAREQLALEHESYEMGEARFRKMFERQLK
SP6 ..........................MQDLHAIQLQLEEEMFNGGIRRFEADQQRQIA
50 60 70 80 90
KP34 NGGIMNLPMTQRMLTSAYEVAAAAIDEMRNVKAPGIGG....KYRRFLRLIPLDVLTTLS
Syn5 RSYASSNIESRKAIATFLDPIAQRIGERLFTLRRGTGAVDAAEVYKHLKNADHHHLALIT
T7 AGEVADNAAAKPLITTLLPKMIARINDWFEEVKAKRGK..RPTAFQFLQEIKPEAVAYIT
SP6 AGSESDTAWNRRLLSELIAPMAEGIQAYKEEYEGKKGR..APRALAFLQCVENEVAAYIT
100 110 120 130 140 150
KP34 LCTMFEAFSVAPGESASRRQTAQAVMSALGRNVQSELLSLQLRNVAPAYMDRVYEY.LTE
Syn5 MKTALDVLGK......DPEPQIQQLTTAIGRNIQLELRLTYYAEENPELYKQASRF.FHA
T7 IKTTLACLTS......ADNTTVQAVASAIGRAIEDEARFGRIRDLEAKHFKKNVEEQLNK
SP6 MKVVMDMLNT........DATLQAIAMSVAERIEDQVRFSKLEGHAAKYFEKVKKS.LKA
160 170 180 190 200
KP34 RRTKSPTHIL..RTL....RASAENVHYGHEPWTNAQNISVGRLLCAAVFETGLFQ....
Syn5 GTGT.......RQKATVIKLKFNREGIEWDQWSRVTCHKVGQWLMLAMADVTGWIERAT
T7 RVGHVYKKA.FMQVVEADMLSKGLLGGEAWSSWHKEDSIHVGVRCIEMLIESTGMV...S
SP6 SRTKSYRHAHNVAVVAEKSVAEKDADFDRWEAWPKETQLQIGTTLLEILEGSVFYNGEPV
210 220 230 240 250 260
KP34 ...WKTGSGNL...SMLYPADDVMEAFQQLV.ESADTVTMKPPMLVPPVQHTTMWDGGYL
Syn5 ....DRTSGGRKTKTRICYSREFLQHRDTILAAAEQLAFCQWPMLCPPIEWSNDHNGGYL
T7 LHRQNAGVVGQ.DSETIELAPEYAEAIATRAGALAGISPMFQPCVVPPKPWTGITGGGYW
SP6 FMRAMRTYGGK.TIYYLQTSESVGQWISAFKEHVAQLSPAYAPCVIPPRPWRTPFNGGFH
270 280 290 300 310 320
KP34 TPIDNRGTYHNSHIDRTRLREVAEAFKSADGIKKALNKAQETPYRINKRILELVQEARAL
Syn5 SEQIRRVNPLIRKTGPLGTRK......QGDIPLAMLNNLQGQAYKVNPEVLDIANHCYES
T7 ANGRRPLALVRTHSK.KALMRY..EDVYMPEVYKAINIAQNTAWKINKKVLAVANVITKW
SP6 TEKVASRIRLVKGNR.EHVRKL..TQKQMPKVYKAINALQNTQWQINKDVLAVIEEVIRL
330 340 350 360
KP34 GIGVGMPRSVPEPKPE..........WYLDG.VPKENYTEEELDRFGEWKTRMSLWYSA
Syn5 NVTVG.....KFIRHAPLPVPPSPGEDC............TEDQLTAYKRARREAEDF
T7 KHC..PVEDIPAIEREELPMKPEDIDM............N...PEALTAWKRAAAAVYRK
SP6 DLGYGVPSFKPLIDKENKPANPVPVEFQHLRGRELKEMLSPEQWQQFINWKGECARLYTA
370 380 390 400 410 420
KP34 DRKRVSQLRSLLTTLEMAEEFKDEKALYFPTCVDWRYRLYFK.SSLHPQGSDLQKALLEF
Syn5 NAQISQKNWRTTEVMYVARKYADEASFWMPASFDYRGRVYFLNTALNPQGTDFDKALLYF
T7 DKARKSRRISLEFMLEQANKFANHKAIWFPYNMDWRGRVYAV.SMFNPQGNDMTKGLLTL
SP6 ETKRGSKSAAVVRMVGQARKYSAFESIYFVYAMDSRSRVYVQSSTLSPQSNDLGKALLRF
430 440 450 460 470 480
KP34 GRGKPL.GDRGLFWLKVHVATCFGYDKTLFEDRAAWVDAN..FAEIEQLTVSPFDCPAFT
Syn5 AEEGPVN....EWWLSFHVATTYGLDKETMVNRVQWARDN..HELIDRIASDPVRHTEWH
T7 AKGKPI.GKEGYYWLKIHGANCAGVDKVPFPERIKFIEEN..HENIMACAKSPLENTWWA
SP6 TEGRPVNGVEALKWFCINGANLWGWDKKTFDVRVSNVLDEEFQDMCRDIAADPLTFTQWA
490 500 510 520 530 540
KP34 SADSPWCLLAAAIDLVNA...VRSGCPEEHISRIPVAMDATNSGGQHLSALLRDPVGGRL
Syn5 DADEPWCFLAACLEYKACVI.....DGTKQTSGLPIGIDATCSGLQHLAAMTRCGRTAAL
T7 EQDSPFCFLAFCFEYAGVQH.....HGLSYNCSLPLAFDGSCSGIQHFSAMLRDEVGGRA
SP6 KADAPYEFLAWCFEYAQYLDLVDEGRADEFRTHLPVHQDGSCSGIQHYSAMLRDEVGAKA

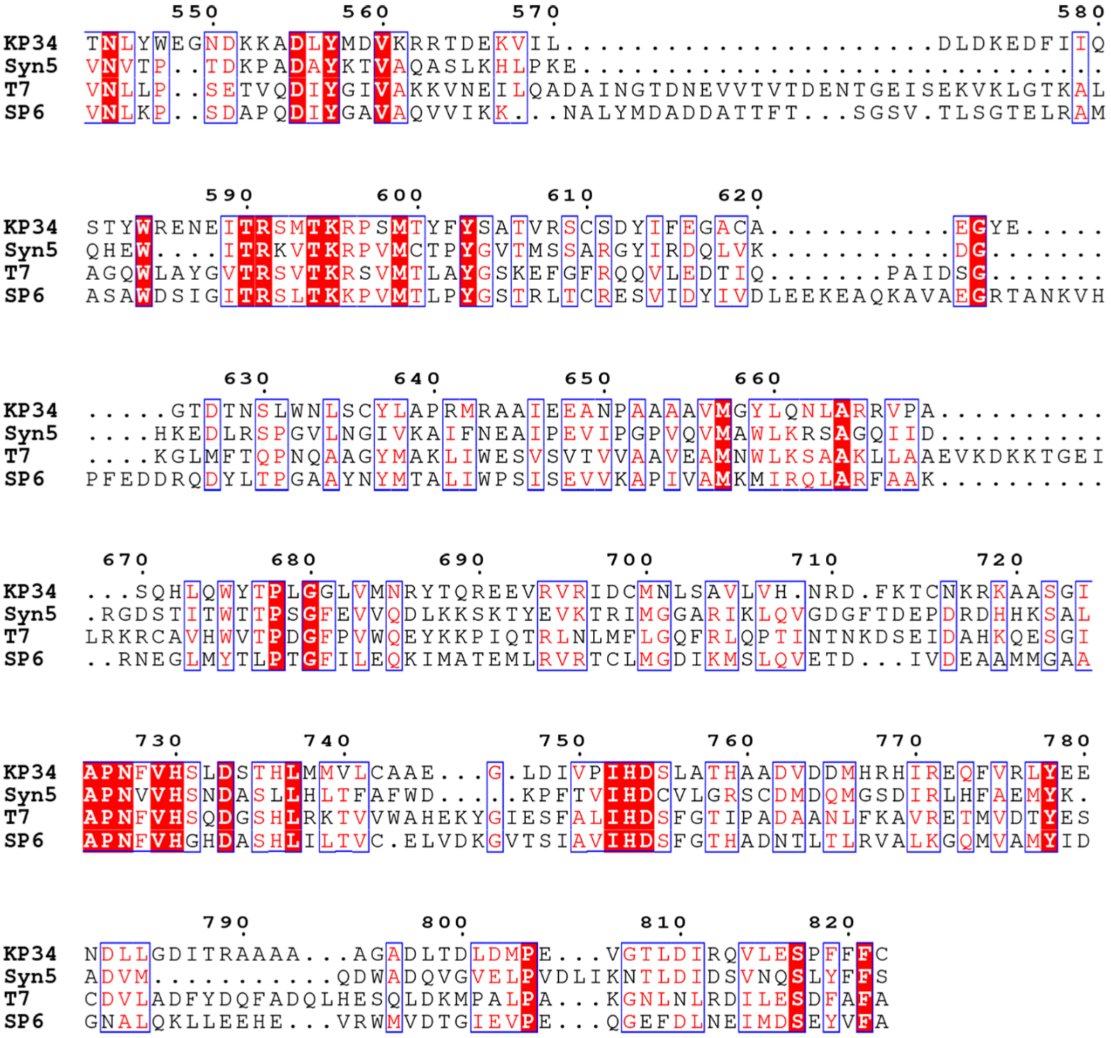

Supplementary Figure 1. Sequence alignment of the KP34 RNAP, Syn5 RNAP, T7 RNAP and SP6 RNAP was performed using Clustal Omega. The alignment was processed for publication using the ESPRIPT server v 3.0. The amino acid sequence of KP34 RNAP shares a similarity of 24.39%, 27.28% and 25.54% with Syn5, T7 and SP6 RNAP, respectively.

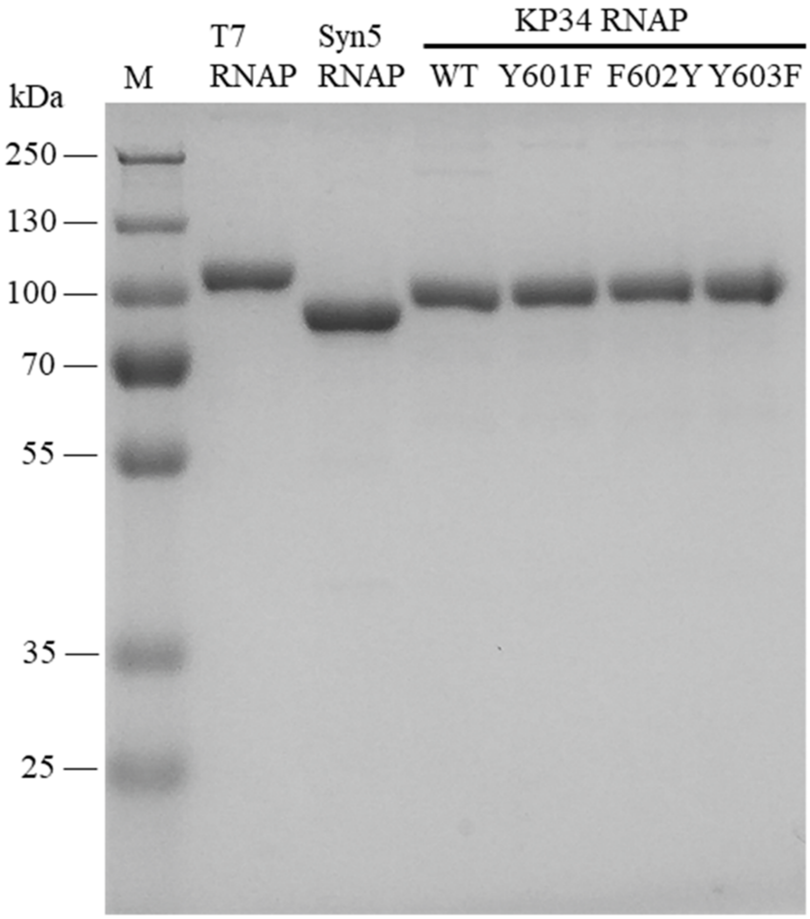

Supplementary Figure 2. SDS-PAGE analysis of all RNA polymerases in this work. All enzymes are N-terminal His-tagged and purified through Ni-NTA-agarose column and gel filtration column. Proteins were stained with Coomassie Blue. M: molecular mass marker.

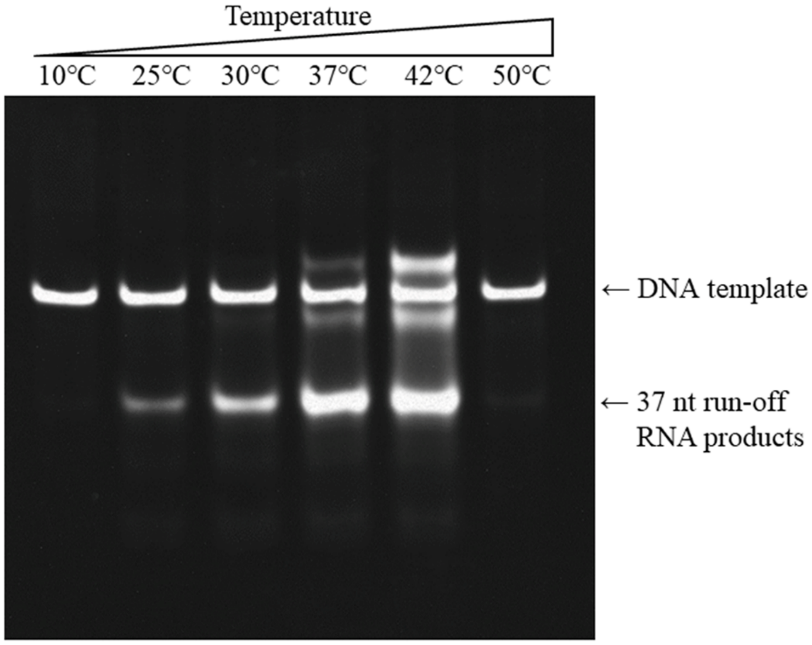

Supplementary Figure 3. Effect of reaction temperature on KP34 RNAP activity. The transcription assays of the KP34 RNAP (1μM) were performed at various temperatures in the presence of 40 mM Tris-HCl (pH 8.0), 20 mM $MgCl_2$, 2 mM spermidine, 20 mM DTT, 4 mM of each of the 4 NTPs and 2 μM DNA template, 40 U/μl RNaseOUT™ Recombinant Ribonuclease Inhibitor, 0.04 U/μl *E. coli* inorganic pyrophosphatase, for 1 h. DNA template and RNA products were separated on a 12% TBE native gel. Various temperatures for the reactions were shown at the top of the gel.

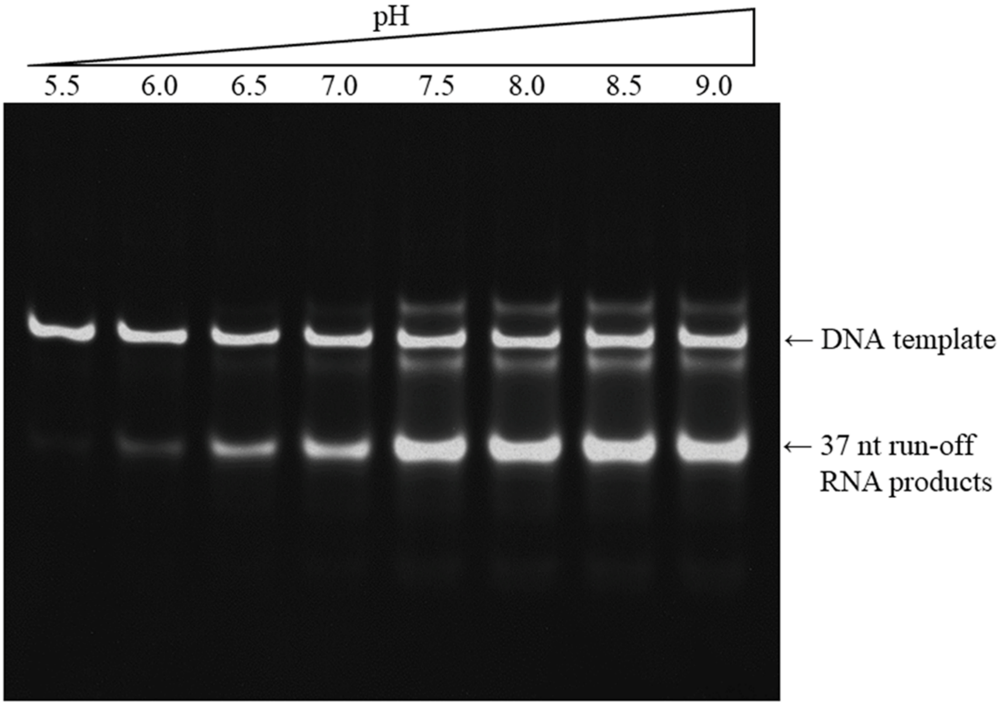

Supplementary Figure 4. Effect of pH on KP34 RNAP activity. The transcription assays of the KP34 RNAP (1μM) were performed using buffers with various pH at 37°C for 1 h in the presence of 20 mM $MgCl_2$, 2 mM spermidine, 20 mM DTT, 4 mM of each of the 4 NTPs and 2 μM DNA template, 40 U/μl RNaseOUT™ Recombinant Ribonuclease Inhibitor, 0.04 U/μl *E. coli* inorganic pyrophosphatase. Three different buffers (all at 40 mM concentrations) were used to prepare different pHs: NaH_2PO_4-Na_2HPO_4 (pH 5.5, pH 6.0, pH 6.5, pH 7.0), Tris-HCl (pH 7.5 and pH 8.0), and Glycine-NaOH (pH 8.5 and pH 9.0). DNA template and RNA products were separated on a 12% TBE native gel. Various pH conditions for the reactions were shown at the top of the gel.

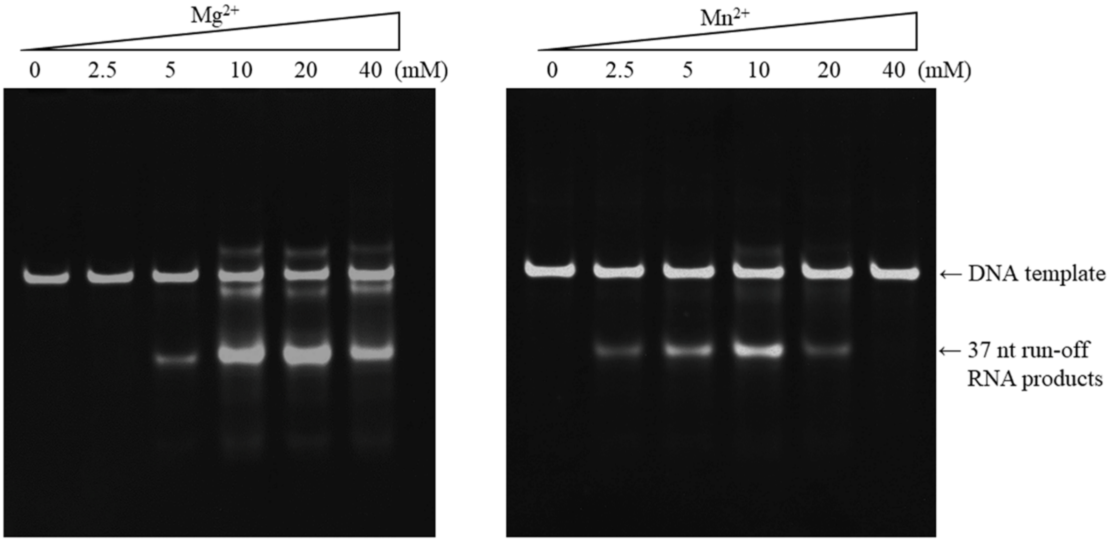

Supplementary Figure 5. Effect of $MgCl_2$ and $MnCl_2$ on KP34 RNAP activity. The transcription assays of the KP34 RNAP (1μM) were performed with Mg^{2+}/Mn^{2+} at 37℃ for 1 h in the presence of 40 mM Tris-HCl (pH 8.0), 2 mM spermidine, 20 mM DTT, 4 mM of each of the 4 NTPs and 2 μM DNA template, 40 U/μl RNaseOUT™ Recombinant Ribonuclease Inhibitor, 0.04 U/μl *E. coli* inorganic pyrophosphatase. DNA template and RNA products were separated on a 12% TBE native gel. Concentrations of Mg^{2+} and Mn^{2+} in the reactions were shown at the top of the gel.

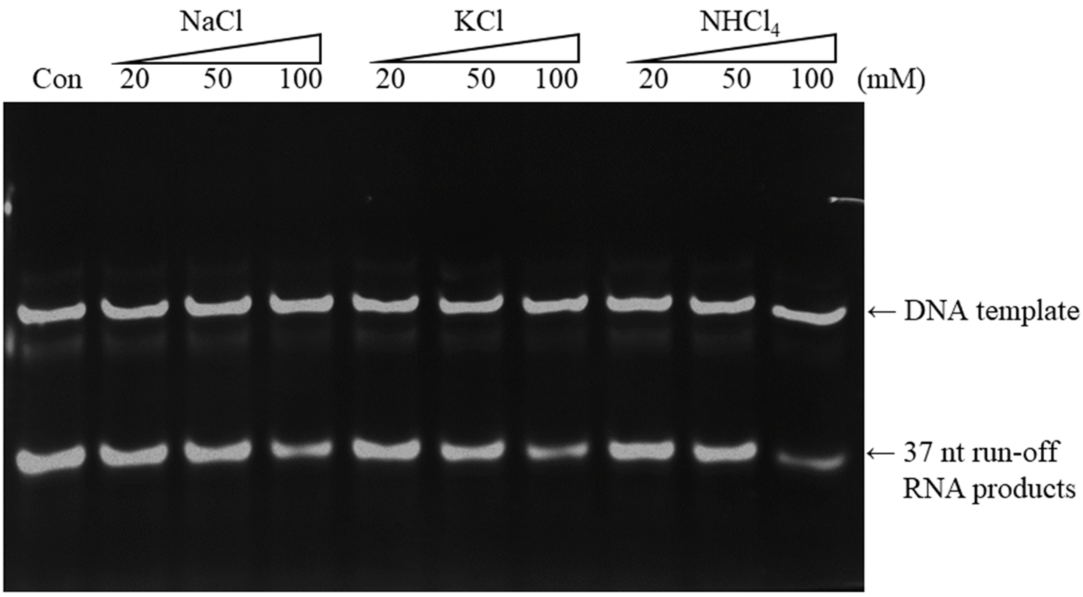

Supplementary Figure 6. Effect of NaCl, KCl and $NHCl_4$ on KP34 RNAP activity. NaCl, KCl or $NHCl_4$ at various concentrations were added to the transcription assays contained 40 mM Tris-HCl (pH 8.0), 2 mM spermidine, 20 mM DTT, 20 mM $MgCl_2$, 4 mM of each of the 4 NTPs, 40 U/µl RNaseOUT™ Recombinant Ribonuclease Inhibitor, 0.04 U/µl *E. coli* inorganic pyrophosphatase, 2 µM DNA template and 1 µM KP34 RNAP. Reaction mixtures were incubated at 37°C for 1 h. DNA template and RNA products were separated on a 12% TBE native gel. Concentrations of NaCl, KCl and $NHCl_4$ in the reactions were shown at the top of the gel. Con: the reaction without salt.

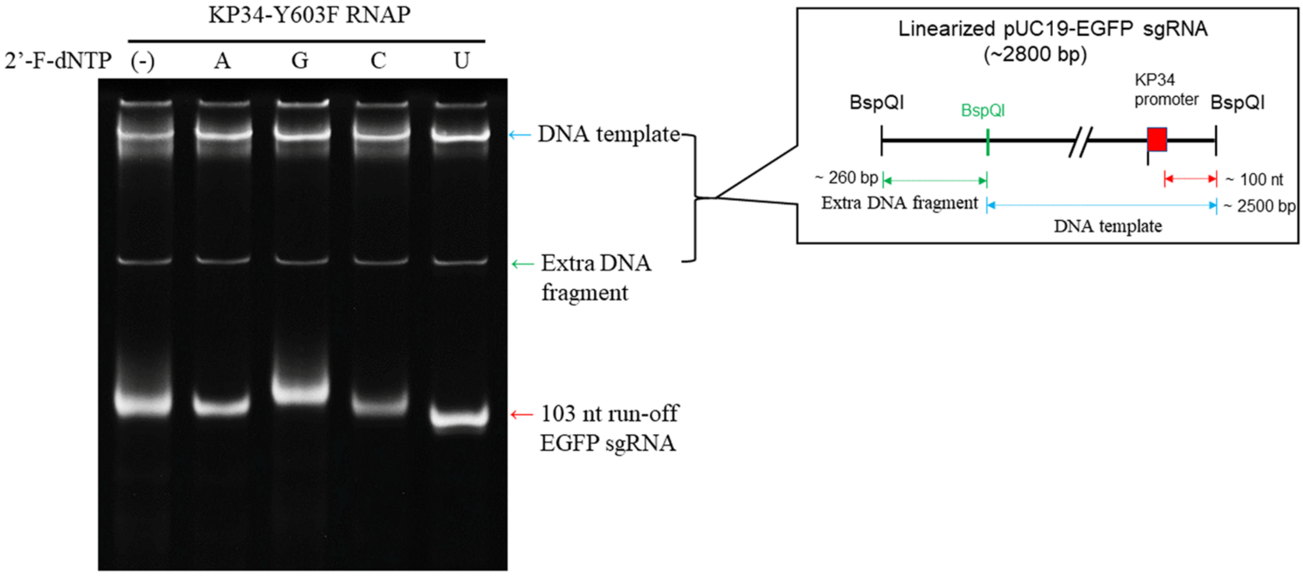

Supplementary Figure 7. Incorporation of 2'-F-dNMPs into the 103 nt sgRNA transcript using KP34-Y603F RNAP. The position of the migration of the DNA template, an extra DNA fragment during template preparation, and the RNA products were marked on the right of the gel. In the reaction, none (-) or one of the four NTPs was replaced by its 2'-F-dNTP analog as indicated at the top of the gel. In the right panel box: schematic showing the preparation of transcription template by digestion of the plasmid using BspQI restriction enzyme.
